# Pattern, severity, and treatment outcomes in acute poisoning patients admitted to the Saint Peter Specialized Hospital Toxicology Center in Addis Ababa, Ethiopia, 2023: a retrospective study

**DOI:** 10.3389/ftox.2025.1517970

**Published:** 2025-02-26

**Authors:** Yared Negussie Kebede, Abdurehman Seid Mohammed, Chekole Sileshi Menberu, Getachew Mekete Diress

**Affiliations:** ^1^ Department of Public Health, Saint Peter Specialized Hospital, Addis Ababa, Ethiopia; ^2^ Department of Anesthesia, School of Medicine, College of Health Sciences, Debre Tabor University, Debre Tabor, Ethiopia

**Keywords:** treatment, outcome, toxicology center, poisoning, Ethiopia

## Abstract

**Background:**

Poisoning is a global public health problem that has more unfavorable outcomes in developing countries. This study aimed to assess treatment outcomes and associated factors among poisoned patients treated at Saint Peter Specialized Hospital Toxicology Center.

**Methods:**

An institutional-based retrospective cohort study was employed by reviewing medical chart records of acutely poisoned patients who had been admitted at St. Peter Specialized Hospital Toxicology Center on 01/01/2017 to 30/12/2023 and the medical chart records review was employed from 01/01/2024 to 30/01/2024. This study analyzed records of 553 poisoned patients. A systematic random sampling technique was used to select the study unit. Data were entered and analyzed using Statistical Package for Social Sciences (SPSS) Windows version 26. A binary logistic regression model was used to identify associated factors for treatment outcomes of poisoned patients. A p-value <0.05 was considered statistically significant.

**Result:**

A total of 553 documents of poisoned patients were assessed. The overall mortality rate was 18 (3.25%), and four patients developed chronic complications. Factor analyses show that arrival to the center before 4 h (AOR = 0.43, P = 0.008) predicted recovery, whereas arrival at the toxicology center after 8 h (AOR = 2.21, P = 0.004), being hypotensive (AOR = 1.85, P = 0.002), needing intubation (AOR = 2.52, P = 0.014), and the presence of two or more complication (AOR = 3.3, P < 0.001) at admission were predictors of mortality.

**Conclusion and Recommendation:**

The mortality rate for poisoned patients was 18 (3.25%). In this study, delayed arrival to the toxicology center, being hypotensive, needing intubation, and the presence of two or more complications at admission were factors associated with the mortality and morbidity of the patients. Establishing a strong referral link between the toxicology center and regional health institutions, ensuring the availability of possible advanced clinical setup early recognition, and aggressively resuscitating critically ill patients will help minimize unfavorable outcomes.

## 1 Introduction

Poison is defined as any substance that can injure, kill, or impair normal physiological function in humans by producing general or local damage or dysfunction in the body by its chemical activity when introduced into the system or applied externally (Singh and Unnikrishnan, 2006; Ramesha et al., 2009).

Poisoning occurs through the absorption of chemical, physical, or organic substances into the body through the gastrointestinal tract, skin, mucosa, respiratory tract, or parentally, causing damage to the cells, tissues, and organs (Aggarwal et al., 2004). Acute poisoning is an injury in which the toxic effects occur almost immediately, usually within hours of the time of exposure, and can result from exposure to excessive doses of any chemicals. Medicines are responsible for most childhood poisonings (Malangu and Ogunbanjo, 2009).

The prevalence and types of poisoning are different across countries. These differences are a result of countries’ industrial development, agricultural activities, local beliefs and customs, and health policies regarding chemical production, distribution, and utilization (WHO, 2015; Esayas Tadesse, 2016; Elhawary et al., 2023; Kasemy et al., 2022).

World Health Organization (WHO) data from 2016 showed that, worldwide, approximately 106,683 people died due to unintentional poisoning (WHO, 2020). There were an estimated 385 million unintentional acute pesticide poisoning (UAPP) cases worldwide, including approximately 11,000 fatalities. The greatest estimated number of UAPP cases was in southern Asia, followed by southeastern Asia and east Africa concerning nonfatal UAPP (Boedeker et al., 2020). In Africa, there were an estimated 7,800 deaths per year as a result of intentional self-poisoning with pesticides. In addition, snakebites cause between 1,400 and 10,000 deaths in eastern Sub-Saharan Africa (WHO, 2015). In Ethiopia, evidence from hospital-based studies revealed that acute poisoning is a common and important clinical emergency with a varying case fatality rate, ranging from 1.5% to 18.6% (Tefera and Teferi, 2020; Getie and Belayneh, 2020).

The treatment outcome of poisoning is determined by several factors, such as age, dose taken, time from exposure to obtaining healthcare, and the previous health status of the patient (Ghasemi et al., 2018; Yaraghi et al., 2018). Some patients, such as those with organophosphate poisoning, can be treated with an antidote. Other poisoning agents may not have a specific antidote. In such cases, treatment consists of supportive care and close follow-up (Bhandari et al., 2018; Maheswari et al., 2016; De Santi et al., 2024). Therefore, early recognition of severely ill patients and the provision of intense care are very important to reduce further complications and death.

Complications after exposure to poisoning agents may occur in many patients. Pulmonary edema, cardiac and respiratory arrest, acute kidney injury, aspiration pneumonia, and neurological complications were identified as complications following acute poisoning (Dayasiri et al., 2017; Farzaneh et al., 2018).

In Ethiopia, acute poisoning is largely the consequence of man-made errors. Personal or other factors related to the management of chemicals, improper use of guidelines, utilization of wrong dosage, or combinations of these factors were identified as major factors contributing to acute poisoning (Desalew et al., 2011). Poisoning would be less often fatal if treated promptly, but in our country, this is difficult to achieve because of different factors. Previous studies at selected hospitals in Addis Ababa show that only a few antidotes were available, such as atropine sulfate, vitamin K, and pyridoxine (Girma, 2017; Melese, 2018).

## 2 Methods

### 2.1 Study area, study design, and study period

An institutional-based retrospective study was conducted at Saint Peter Specialized Hospital Toxicology Center in Addis Ababa, Ethiopia, from 01/01/2017 to 30/12/2023. St. Peter Specialized Hospital was established in 1963 as a TB sanatorium and gradually expanded to include many other disciplines in recent years. Its services include internal medicine, surgery, pediatrics, gynecology and obstetrics, maternal and child health, psychiatry, dentistry, radiology, voluntary counseling, testing, antiretroviral treatment, dermatovenereology, and toxicology. It has a well-established research department serving investigators from the hospital staff and other institutions and is one of the referral hospitals under the Ministry of Health, in Addis Ababa. The toxicology center at this hospital opened in April 2017 and is a major referral center for poisoning. This toxicology center delivers poison information, clinical service, and training programs to deal with various poisoning-related problems. The setup includes a toxicology ICU with six beds, each with a mechanical ventilator and other critical care equipment. Toxicology emergency has facilities for decontamination and four dialysis machines, and toxicology recovery has four beds for less severely ill patients.

### 2.2 Source and study population

Poisoned patients who had been admitted at St. Peter Specialized Hospital were the source population for this study, and poisoned patients who had been admitted during the study period were the study population.

### 2.3 Inclusion and exclusion criteria

The study included all poisoned patients who were admitted to the emergency department of the toxicology center with a diagnosis of acute poisoning and whose charts were complete and available during data collection time. This encompasses a wide range of poisoning cases, regardless of the substance involved or the severity of the condition. Patients were excluded from the study if their medical records contained incomplete information.

### 2.4 Sample size determination and sampling procedure

For the first objective, a single population proportion formula was used to calculate the sample size by considering the following statistical assumptions.

Z α/2 = the corresponding Z score of 95% CI (confidence interval) = 1.96 d = Margin of error (5%) = 0.05

n = required sample size.

P = proportion of poisoned patients who died = 0.086 (Desalew et al., 2011).

For the second objective, the double population proportion formula was used to calculate the sample size by considering the following statistical assumptions:

$$n=\left(\text{Za}/2+Z\;\right)2\left(p\;\left(1-p\;\right)+p\;\left(1-p\;\right)\right)/(p\;p\;2\text{ Za}/2=\text{desired level of significance }95\%.=1.96$$

ZB = desired power, 80% = 0.84.

#### 2.4.1 Outcome in men and women

P1 = 0.12 (proportions of patients who died among exposed men).

P2 = 0.02 (proportion of patients who died among exposed women).

#### 2.4.2 Outcome among poisoning agents

P1 = 0.2 (proportion of patients who died from organophosphate poisoning).

P2 = 0.215 (proportion of patients who died from phenobarbitone poisoning) (Table 1).

**TABLE 1:** Sample size for factors associated with treatment outcomes of poisoned patients treated at St. Peter Specialized Hospital Toxicology Center from 01/01/2017 to 30/12/2023.

Factors	Sample size	Reference
Sex	93	Desalew et al. (2011)
Poisoning agents	527	Desalew et al. (2011)

Then, the sample size would be the largest sample size of the given double proportion of the variable 527. After adding a 10% contingency rate, the final sample size was 580.

Finally, a simple random sampling technique was used to obtain the required sample size.

### 2.5 Study variables

#### 2.5.1 Dependent variable

Treatment outcome: death or recovered without complication.

#### 2.5.2 Independent variables

Socio-demographic characteristics: age, sex, place of residence, marital status, occupation, and level of education.

Poisoning agents: pesticide, bleaching agents, drug overdose, and carbon monoxide.

Exposure-related factors: place where he/she was exposed, exposure circumstance, route of exposure, pre-hospital care given, mode of visit, and elapsed time.

Clinical characteristics: poisoning severity score at admission, psychiatry history, pregnancy, pre-existing illness, complication at admission, and length of stay.

Treatment given: supportive care, decontamination, and antidote elimination.

### 2.6 Data collection procedures and quality assurance

The checklist was adapted from the international program on chemical safety assessment tools. Data were collected by trained data collectors using a structured questionnaire. Medical records were used to obtain the required data. Data captured include socio-demographic characteristics, poisoning agents, exposure-related factors, clinical characteristics, treatment given, and treatment outcome of the patient.

Two days of training were given to three data collectors with an academic background of a BSC degree in nursing and one coordinator working in the hospital concerning the data collection tool and data collection process before the actual data collection period. In addition, data quality was assured by designing a proper data abstraction tool. The data form was pretested on 5% of the sample size at Saint Paul Hospital Millennium Medical College (SHMMC) to ensure the questions were balanced, correctly constructed, and able to obtain crucial information. The adapted checklist was evaluated by experienced researchers and trained toxicology center staff. Data completeness and consistency were examined by the principal investigator through spot checks and review of the questionnaire. It was internally consistent with a Cronbach’s alpha coefficient of P = 0.72.

### 2.7 Data management and analysis

After data were collected, each questionnaire was cleaned and coded separately by using Epi-info version 7 then exported to and analyzed using SPSS version 26 statistical software. Demographic details of patients included in the analysis were described using simple descriptive statistics. Continuous variables were expressed as means and standard deviations and categorical variables with frequency and percentages. The findings of the study were presented in the form of text, tables, and graphs.

A binary logistic regression model with a 95% confidence interval was used to infer the association between treatment outcome and associated factors. Multivariable logistic regression analysis was performed to control for possible confounders. Multicollinearity among selected independent variables was checked by using the variance inflation factor, and none was found (variance inflation factor (VIF) = 1.86). All the independent variables with p-value <0.25 in bi-variable analysis were included in multivariable analysis to identify predictors for treatment outcome. A p-value <0.05 was considered statistically significant. The assumption of the fitness of goodness in the final model was checked by the Hosmer–Lemeshow test and was found to be fit (P = 0.07).

### 2.8 Ethics consideration

Ethical clearance and ethical approval were obtained from the Institutional Review Committee of Saint Peter Specialized Hospital, with the ethical reference number V111/01/06/2023. The Institutional Review Board (IRB) was waived for consent to participate in the research because the retrospective study could be done by medical record chart review without contacting the patients. A formal letter was submitted to the hospital’s administrators to obtain permission for data collection. The Declaration of Helsinki was considered, and principles and recommendations were used.

### 2.9 Operational definitions

Poisoning severity score: A standardized and generally applicable scheme for grading the severity of poisoning.

Substance use: Use of at least any one of the following substances: alcohol, khat, cigarette, shisha, hashish, or drugs that are assumed to affect the level of thinking and increase the risk of being involved in risky behaviors.

Acute poisoning case: Patient charts diagnosed by the attending physician as acute poisoning cases.

Intentional poisoning: The result of a fully aware person using drugs or giving them to someone else with the intent to harm.

Unintentional poisoning: The result of a person ingesting poison accidentally due to impaired self-awareness brought on by co-occurring mental illnesses or addiction or as a result of an unknown exposure.

Mild: Minor, transient, and spontaneously resolving symptoms.

Moderate: Pronounced or prolonged symptoms.

Severe: Any life-threatening symptoms

Treatment outcome of poisoning: Patient status following treatment, including recovery and death or complications.

Recovered: Poisoned patients improved after treatment.

Death or complication: Death or sequela in patients related to poisoning during treatment.

Antidote: An antidote for each type of poisoning chemical.

Clinical outcome: The state of poisoned patients after they have been treated in the emergency department, reported as survival, death, or complication.

Hypotension: Low blood pressure is when the pressure of blood in the body is lower than normal.

Organ failure: When an organ or organs in the body are no longer able to function properly.

Complication: An unexpected or undesired outcome that occurs during or after a medical intervention or disease that can make a situation more difficult.

Pre-existing illness: A health problem like asthma, diabetes, hypertension, or cancer that started before the date of the poisoning event.

Psychiatric illness: A medical condition that involves a significant disturbance in a person’s thinking, emotions, or behaviors. These disturbances can cause distress or impairment in important areas of life, such as social, occupational, or interpersonal functioning.

Aspiration: A medical condition that occurs when food, liquid, or other material accidentally enters the lungs or airway.

Severity score at admission: Reflects the severity of a patient’s condition when they are admitted to the toxicology center.

Lab abnormalities: Results that lie outside the laboratory reference ranges are considered to be abnormal.

## 3 Results

### 3.1 Socio-demographic characteristics

The records of 553 poisoned patients were selected and reviewed with a response rate of 95.3%. The mean age of patients was 25.25 ± 11.75 years (range from 2 to 80 years), and about 462 (84.7%) of patients were in the age range of 15–44. In the sample, 51 (9.2%) were younger than 15, and more than half of the patients were women. Regarding the place of residence, 36.7% of patients come from outside Addis Ababa. Sixty-five percent of patients were single. Approximately 90% of patients had secondary or lower levels of educational status. The majority of poisoned patients were students (Table 2).

**TABLE 2:** Socio-demographic characteristics of poisoned patients who were treated at St. Peter Specialized Hospital Toxicology Center from 01/01/2017 to 30/12/2023 (n = 553).

Variables	Variable category	Frequency	Percent
Age	<15	51	9.2
	15–29	350	63.3
	30–44	112	20.3
	>44	40	7.2
Sex	Male	241	43.6
	Female	312	56.4
Place of	Addis Ababa	350	63.3
Residence	Outside Addis Ababa	203	36.7
Marital status	Single	348	62.9
	Married	177	32
	Widowed or divorced	28	5.1
Occupation	Employed	170	30.7
	Farmer	45	8.2
	Housewife	30	5.4
	Student	218	39.4
	Daily laborer & No of jobs	90	16.3
Level of education	No formal education	123	22.4
	Primary	199	35.9
	Secondary	121	21.8
	Diploma and above	110	19.9

### 3.2 Poisoning agents that exposed patients exposed to

Of 553 poisoned patients, 203 (36.8%) were poisoned by pesticides, and 90 (16.3%) were poisoned by bleaching agents Table 3. Other identified poisons include formalin, ART, snake bite, alcohol intoxication, food poisoning, and multiple drugs if patients are exposed to more than one drug. Pesticides were the most common agents in men [110 (46%)], and bleaching agents [65 (21.1%)] and analgesic agents [80 (14.6%)] were the most frequent agents in women.

**TABLE 3:** Percentage of patients who were exposed to different poisoning agents.

Variables	Frequency	Percentage
Pesticide	203	36.8
Bleaching agent	90	16.3
Analgesic	80	14.6
Carbon monoxide	51	9.3
Multiple drugs	51	9.3
Anti-epileptic	23	4.2
Anti-psychotic	13	3.3
Traditional medicine	14	2.6
Others	28	5.1

**FIGURE 1:**
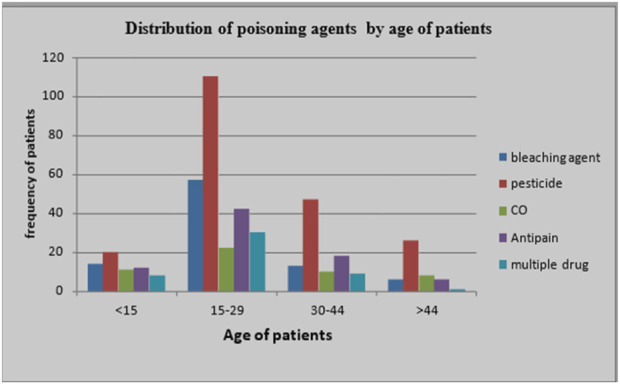
Poisoning agent distribution by age of poisoned patients treated at St. Peter Specialized Hospital Toxicology Center from 01/01/2017 to 30/12/2023.

Bleaching agents, anti-pain, and carbon monoxide were the most common agents in patients from within Addis Ababa, whereas pesticides are the most frequently ingested substance by patients from outside Addis Ababa. Jobless, daily laborers, and students commonly ingest pesticides (Figure 1).

### 3.3 Exposure-related factors

Concerning exposure-related factors, 454 (82.1%) of patients were exposed at home, 41 (7.4%) at a shop, and 58 (10.5%) at other places (workplace, industry, and health institution). Exposure circumstances included intentional/suicidal exposure [458 (82.5%)], accidental [92 (16.5%)], and unspecified [3 (1%)]. Among all patients, 467 (84.5%) were exposed by ingestion, 55 (9.9%) inhaled the substance, and 31 (5.6%) were exposed through other means (cutaneous, bite, and injection). Only some patients obtained pre-hospital care, such as drinking water, milk, or juice. Regarding patients’ mode of visit, 156 (28.9%) were self-visit, and 397 (71.9%) visited by referral. Elapsed time ranged from 20 min to 4 days with a median time of 4 h Table 4.

**TABLE 4:** Exposure-related factors and clinical characteristics of poisoned patients treated at St. Peter Specialized Hospital Toxicology Center from 01/01/2017 to 30/12/2023 (n= 553).

Variable	Variable category	Frequency	Percentage (%)
Place of exposure	Home	454	82.1
	Shop	41	7.4
	Other places	58	10.5
Exposure circumstance	Intentional	458	82.5
	Accidental	92	16.5
	Unspecified	3	1
Exposure route	Ingestion	467	84.5
	Inhalation	55	9.9
	Other	31	5.6
Mode of visit	Self-visit	156	28.9
	By referral	397	71.1
Elapsed time in	<240 m	265	47.9
	240–480 m	217	39.3
	>480 m	71	12.8
Psychiatry	Yes	113	20.6
	No	440	79.4
Pregnancy	Yes	64	11.57
	No	489	88.43
Pre-hospital care given	Yes	92	16.7
	No	461	83.3
Pre-existing illness	Yes	175	31.7
	No	378	68.3
Severity score at admission	Mild	208	37.6
	Moderate	217	39.2
	Severe	128	23.2
Complication at admission	Yes	133	24.1
	No	420	75.9
Aspiration pneumonia	Yes	127	22.9
	No	426	77.1
Lab abnormalities	Yes	225	40.6
	No	328	59.4
Hypotension	Yes	131	23.7
	No	422	76.3
Need for intubation	Yes	229	41.4
	No	324	58.6
Organ failure	Yes	129	23.3
	No	424	76.7

### 3.4 Clinical characteristics of poisoned patients

Patients’ clinical characteristics were assessed by using severity scores at admission, complications, and length of stay. The present study revealed that based on poisoning severity score, 208 (37.6%) patients had mild, 217 (39.2%) patients had moderate, and 128 (23.2%) patients had severe poisoning severity scores at admission. There were 113 (20.6%) poisoned patients who had a psychiatry history. Approximately 175 (31.7) of the patients in the study had different pre-existing illnesses Table 4. Among poisoned female patients, 64 (11.57%) were pregnant. Poisoning can be the cause of many severe complications. Complications at admission were observed in 133 (24.1%) patients, and the most common were hypotension [131 (23.7%)] followed by the need for intubation [229 (41%)] Table 4. The median length of stay in the toxicology center was 48 h, and half of the patients were discharged within 48 h.

### 3.5 Treatment approaches for poisoned patients treated at St. Peter Toxicology Center

Supportive treatment was given to 508 patients (92%), antidotes were given to 154 (28%) patients, 420 (76%) patients required decontamination (GI, skin, and ocular), and two patients were treated with hemodialysis for elimination.

Treatment outcome of poisoned patients treated at St. Peter Specialized Hospital Toxicology Center: Among all (553) poisoned patients, 18 (3.25%) of them died, and four patients recovered with complications (two patients were oxygen-dependent, one had a neurologic disorder, and one had anoxic brain damage. Most [531 (96.1%)] patients recovered without complications.

### 3.6 Factors predicting treatment outcome of poisoned patients

In a bi-variable analysis, variables with a p-value of <0.25 included anti-pain, elapsed time, mode of visit, pre-existing illness, severity score at admission, presenting with hypotension, need for intubation, and two or more complications (Table 5).

**TABLE 5:** Bi-variable and multivariable logistic regression analyses of predictor factors for treatment outcome of poisoned patients treated at St. Peter Specialized Hospital Toxicology Center from 01/01/2017 to 30/12/2023 (n = 553).

Variable	Variable category	Treatment outcome		COR (95% CI)	AOR (95% CI)	p-value
		Recovered	Death			
Mode of visit	Self-visit	156	9	1.25 (0.01–2.81)	2.39 (0.07–11.96)	0.21
	By referral	397	13	1	1	1
Lab abnormalities	Yes	36	7	1.41 (1.03–6.78)	1.42 (0.33–4.58)	0.27
	No	465	15	1	1	1
Severity score at admission	Mild	206	0	1	1	1
	Moderate	217	3	1.43 (1.32–4.76)	1.85 (0.41–17.26)	0.59
	Severe	128	19	2.2 (0.34–5.96)	3.46 (0.81–10.34)	0.07
Hypotension	Yes	25	14	1.53 (1.04–6.8)	1.85 (1.22–5.8)	0.002**
	No	526	8	1	1	1
Organ failure	Yes	4	12	2.37 (2.14–7.8)	2.31 (0.09–8.73)	0.08
	No	527	10	1	1	
Need for intubation	Yes	11	18	1.42 (1.4–7.96)	2.52 (1.02–8.77)	0.014*
	No	520	4	1	1	1
Analgesia	Yes	74	6	1.46 (0.06–9.83)	1.46 (0.6–9.83)	0.07
	No	457	16	1	1	1
Two or more complications	Yes	2	11	2.32 (2.01–7.52)	3.30 (2.32–7.7)	0.001**
	No	529	9	1	1	1
Elapsed time	<240 m	265	2	1	1	1
	240–480 m	217	6	0.4 (0.13–0.74)	0.43 (0.35–0.82)	0.08
	>480 m	70	14	1.37 (1.09–6.69)	2.21 (1.29–6.85)	0.004**
Pre-existing illness	Yes	166	9	1.48 (0.27–6.47)	2.19 (0.08–12.6)	0.64
	No	369	13	1	1	1

*, **, statistically significant (p-value <0.05).

COR, crude odds ratio, AOR, adjusted odds ratio, CI, confidence interval, I, reference.

Multivariable logistic regression identified delayed arrival to the toxicology center, hypotension, need for intubation, and two or more complications at admission as risk factors for death in poisoned patients.

Using multivariable analysis, the first factor identified in this study was elapsed time (the time interval between exposure to poisoning agents and arrival at the toxicology center).

The odds of death in patients who arrived at the toxicology center within 4 h were 57% less (AOR = 0.43, CI: 0.35–0.82) than in patients who arrived between 4 h and 8 h.

The odds of death in patients who arrived at the toxicology center after 8 h were 2.2 times (AOR = 2.21, CI: 1.29–6.89) as high as the odds of those who had arrived between 4 h and 8 h.

The odds of death in patients who had hypotension at admission were twice the odds of (AOR = 1.85, CI: 1.22–5.8) of those who had no hypotension.

The odds of death/complication for patients who needed intubation at admission were 2.5 times the odds of (AOR = 2.52, CI: 1.02–8.77) of those who did not need it.

The odds of death in patients who presented with two or more complications were three times the odds (AOR = 3.30, CI: 2.32–7.70) of patients who did not have two or more complications.

## 4 Discussion

Among poisoning patients treated at Saint Peter Toxicology Center, the death rate was (3.25%). Since the kind of poison and the corresponding morbidity and mortality vary from one place to another and might change over time, poisoning is a major problem in developing countries. Assessing poisoning prevention and treatment strategies involves figuring out treatment results and examining the factors.

In the current study, more than half of poisoned patients were female (56.4%). This result supported the International Poison and Control Program's claims that pesticide poisoning is the most common cause of death worldwide in regions that are dominated by agriculture. Iran also observed high female predominance (68.2%) (Ghasemi et al., 2018; Desalew et al., 2011; Adinew et al., 2017). The predominance of young age was observed in poisoning exposure. The probable reason for this is that these young adults face different stressors and have poor coping mechanisms. The predominance of young age (working age groups) is consistent with findings of studies conducted elsewhere in Gondar (Ethiopia), Zambia, South Africa, Nepal, and India (Bhandari et al., 2018; Maheswari et al., 2016; Z’gambo et al., 2016).

In the current study, pesticides were the most common poisoning agent. The sub-group of pesticides most frequently ingested pesticide was organophosphorus, followed by rodenticide. This finding supported the report by the International Poison and Control Program that pesticide poisoning is a major cause of death in agriculture-dominated areas in the world (WHO, 2021). The second most employed agent was bleaching agents. This is probably due to their availability and the belief that these agents kill in a shorter time with less suffering. Africa, including Ethiopia, is a region that reported the highest mortality due to pesticide poisoning (WHO, 2015; Chelkeba et al., 2018).

Another finding not seen in the previous studies is the increased use of pain medication as a poisoning agent. The reason may be poor implementation of policies on over-the-counter drugs, increased incidence of chronic illness, and easy availability of drugs. The finding shows that while there is a decline in suicide by psychiatric drugs, these patients ingest bleaching agents and pain medication. This may be due to increased community awareness of the need to store psychiatric medications safely.

Concerning circumstances of poisoning, more than 80% of patients were exposed intentionally/suicidal, and most of those people were women. This may be due to their higher socio-economic burden. The frequency of intentional poisoning was significantly higher in adults aged 15–29 (54.7%) years. This finding aligned with the WHO Report 2012 that shows, among young adults in the age group 15–29 years, suicide accounts for 8.5% of all deaths and is the second leading cause of death in the age group after road traffic injuries (Varghese et al., 2017).

Suicide by poisoning in young adults is an important concern that requires public health interventions. Another issue is that psychiatric patients are not sent to health institutions for various reasons, such as a belief that psychiatric illness is untreatable and poor health facilities for mental health (Gualano et al., 2021). Such patients are obliged to stay at home in the worst conditions, and the caregivers may be children or older people who are unable to care for these patients. Suicidal ideations and attempts may be due to these factors. Our study demonstrated that most ingested substances in this category were bleaching agents (31.5%). If psychiatric patients are cared for at home, such dangerous chemicals should be placed out of their reach.

Unintentional poisoning was included in the SDG as target 3.9, and the WHO Report 2012 was used as a baseline. The report identified that the mortality rate due to unintentional poisoning was higher in children under the age of five and adults above age 50 (Varghese et al., 2017). This study’s findings partially supported this report. The reason behind this may be age-related susceptibility, and it is common to see that these age groups are employed in highly risky working areas.

Findings from this study supported the literature on the fact that elapsed time was a determinant of treatment outcome. The odds of death/complication in patients who arrived at the toxicology center after 8 h were 2.2 times the odds of those patients who had arrived between 4 h and 8 h. More than 50% of patients arrived at the toxicology center after 4 h. Some reasons for the delay include distance from the hospital (outside the city), poor referral systems, and a belief in traditional management.

The findings of this study show that patients who arrived early were more likely to have a chance of recovery than those who arrived late. This was consistent with previous findings of a study at Jimma Medical Center, which showed that poisoned patients who arrived too late were less likely to achieve successful treatment outcomes (Ahmed et al., 2018). This also corresponds to a study conducted in Korea and England, which found that patients who arrived within 1 h had successful treatment outcomes (Sun et al., 2016). Based on the findings of this study, poisoned patients are encouraged to seek health services early.

Triage, which involves measuring vital signs, is one important step in treating poisoned patients (Aggarwal et al., 2004; Sharif et al., 2023). Studies also supported the fact that triage parameters were important outcome predictors of poisoned patients. Identifying clinical statuses at admission is a guide to the intervention that the patient will be provided. In this study, patients who presented without complications had a higher chance of recovery than those presenting with complications.

One complication observed in the current study was hypotension. Based on this study, the odds of death in patients who had hypotension at admission were twice the odds of those who did not have hypotension. This finding is in line with previous findings in studies conducted in Nepal, Iran, and Egypt (Ghasemi et al., 2018; Bhandari et al., 2018; Lashin et al., 2024a).

Thirty patients were intubated in the current study, and 36.4% of them passed away. Patients who required intubation upon entry had 2.5 times the risk of dying or experiencing a problem compared to those who did not. According to the study’s findings, the highest fatality rate was linked to poisoned individuals who required intubation. This result corroborated other study findings (Farzaneh et al., 2018; Carroll et al., 2013; Lashin et al., 2024b; Abd Elghany et al., 2023).

We did not find significant differences in the outcome concerning different types of poisoning agents, pre-existing illness severity score at admission, mode of visit, organ failure at admission, and abnormal lab results. Further coordinated studies on detailed clinical parameters should be conducted to deeply understand the pathology of poisoning, design appropriate interventions, and minimize such unacceptable mortality.

## 5 Conclusion and recommendations

The mortality rate for poisoned patients treated at Saint Peter Toxicology Center was higher than WHO-recommended acceptable mortality (1%–2%). In this study, delayed arrival to the toxicology center, being hypotensive, the need for intubation, and the presence of two or more complications at admission were factors associated with unsuccessful treatment outcomes. Ensure availability of possible advanced clinical setup and antidote because poisoned patients may present with severe complications. Early recognition and aggressive resuscitation of patients are mandatory.

## 6 Strengths and limitations of the study

This study included epidemiological and clinical factors not addressed in previous research. Its findings generated baseline information on the treatment outcomes of poisoned patients and identified factors associated with the treatment outcomes.

The limitation regarding this study’s findings was that this toxicology center opened in 2017, a relatively short period to show any trends in poisoning.

## Data Availability

The datasets presented in this study can be found in online repositories. The names of the repository/repositories and accession number(s) can be found in the article/Supplementary Material.

## Abbreviations

ABC, Airway breathing circulation; AOR, Adjusted odds ratio; BSC, Bachelor of science; CI, Confidence interval; COR, Crude odds ratio; CO, Carbon monoxide; GCS, Glasgow coma scale; GI, Gastrointestinal; HMIS, Health management information system; Lab, Laboratory; PI, Principal investigator; SDG, Sustainable Development Goal; SPSS, Statistical Package for Social Science; VIF, Variance inflation factor; WHO, World Health Organization.
