# Supplementary material for: Pattern, severity, and treatment outcomes in acute poisoning patients admitted to the Saint Peter Specialized Hospital Toxicology Center in Addis Ababa, Ethiopia, 2023: a retrospective study
Source: Front Toxicol. 2025 Feb 26;7:1517970. doi: 10.3389/ftox.2025.1517970 (PMC11897292; doi:10.3389/ftox.2025.1517970)
Supplement: Supplementary file 1 [file Table1.DOCX]

According to our result, the overall incidence of Post-Dural Puncture Headache (PDPH) was 39.2 %( **Figure-1).**

**Factor associated with Post-Dural Puncture Headache (PDPH) after spinal anesthesia among surgical patients.**
